# Association between Adherence to the Mediterranean Diet and Physical Fitness with Body Composition Parameters in 1717 European Adolescents: The AdolesHealth Study

**DOI:** 10.3390/nu12010077

**Published:** 2019-12-27

**Authors:** Pablo Galan-Lopez, Antonio J. Sanchez-Oliver, Maret Pihu, Thórdís Gísladóttír, Raúl Domínguez, Francis Ries

**Affiliations:** 1 Department of Physical Education and Sports, Faculty of Educational Sciences, University of Seville (Research Lab HUM 962: Sports and Society), 41013 Sevilla, Spain; pgalan1@us.es (P.G.-L.); fries@us.es (F.R.); 2 Department of Human Motricity and Sports Performance, University of Seville, 41013 Seville, Spain; sanchezoliver@us.es; 3 Institute of Sport Sciences and Physiotherapy, Faculty of Medicine, University of Tartu, 51005 Tartu, Estonia; maret.pihu@ut.ee; 4 Research Center for Sport and Health Science, School of Education, University of Iceland, 105 Reykjavík, Iceland; thg@hi.is; 5 Faculty of Health Sciences of Universidad Isabel I, Universidad Isabel I, 09004 Burgos, Spain

**Keywords:** physical fitness, dietary patterns, risk and protective factors, Mediterranean diet, adolescents

## Abstract

Obesity, low levels of physical fitness, and unhealthy eating patterns are responsible for part of the health problems of adolescents today. The current study aimed at examining the association between the adherence to the Mediterranean diet (MD), through each answer to the items of the Adherence to the MD Questionnaire (KIDMED), and physical fitness with body composition parameters (body mass index (BMI), percentage of body fat, and waist circumference) in 1717 European adolescents (N = 900 boys, N = 817 girls). Data of body composition, physical fitness results, and the answers to KIDMED were analyzed by the Student’s *t*-test. Additionally, the effect size (ES) was calculated and a Chi-square test analyzed the proportion of participants with and without over waist circumference, overfat, and overweight in each KIDMED question. The relative risk of suffering over waist circumference, overfat and overweight in relation to the responses was calculated by Odd-Ratio. Adherence to the MD did not influence the condition of over waist circumference, overfat and overweight, although certain dietary habits were identified as risk factors for their development. Over waist circumference, overfat, and overweight boys and girls presented higher levels of body mass, waist circumference, body fat percentage, and BMI (*p* < 0.001; ES = 1.73–3.38), as well as lower levels of all the parameters of the physical fitness analyzed (*p* < 0.001; ES = 0.45–1.08), except the handgrip test. A direct relationship between fitness and over waist circumference, overfat, and overweight was found.

## 1. Introduction

The global prevalence of overweight and obesity in children and adolescents has reached worrying levels [1,2]. According to official data from the World Health Organization (WHO), in 2022 there will be more children and adolescents with obesity than with underweight. The global rates of obesity of the child and adolescent population have multiplied by 10 worldwide in individuals from 5 to 19 years of age and increased from 11 million in 1975 to 124 million in 2016. In addition, although they did not reach the threshold of obesity, 213 million of this population presented overweight in 2016 [3].

Predictive obesity indices are commonly based on the body mass index (BMI), which, despite being widely used, does not provide a real measure of obesity [4]. As abdominal fat especially has a great impact on health [5], it is important to include other forms of assessing obesity that take this parameter into account. Therefore, it is essential to complement the BMI assessment with both, a record of body fat percentages and the waist circumference [4].

Healthy habits are a fundamental part in the prevention and intervention of obesity, and public health organizations pursue them in order to promote health among children and adolescents [6]. Physical activity (PA) and the adoption and maintenance of healthy eating patterns are crucial for a good health-related quality of life [7,8].

Physical inactivity and low levels of physical fitness (PF) are documented risk factors for obesity even at early ages [9]. In contrast, high levels of PA and PF largely neutralize the adverse effects of excess adiposity and other risk factors related to obesity [10,11]. Without a minimum level of daily PA, there is a greater probability of children and adolescents having a lower quality of life than their parents had [12]. However, currently, PA and PF levels in children and adolescents are alarmingly low [13]. Thus, the assessment and control of PF are crucial to identify and to establish appropriate public health strategies for these ages [14].

Inadequate nutrition is related to a higher prevalence of obesity in children and adolescents [15]. On the contrary, a healthy eating pattern, such as the Mediterranean diet (MD), is considered as fundamental for preventing and reducing obesity at these ages [16]. The MD can be considered as the most studied and known dietary pattern in the world [17] and the Adherence to the Mediterranean Diet Questionnaire (KIDMED) is the most used validated instrument to measure the adherence to the MD in children and adolescents [18]. The combination of Mediterranean cooking techniques, food, nutrients, and lifestyle interact in a way that makes the MD a powerful preventive tool and directly applicable for improving the quality of life and reducing diseases [17], as well as for increasing life expectancy [19].

Given the value of an adequate diet and a suitable level of PF for preventing obesity and shaping health-related quality of life in adolescents, each question of the KIDMED questionnaire was analyzed separately by the authors in order to see their relationship with the anthropometric aspects that can determine overweight or obesity. Doing this a novel character to the relationships established between the KIDMED questionnaire and body composition in the existing literature is added. Therefore, the present study has the following objectives: (1) to analyze the association between the MD, evaluating each of the answers of the KIDMED questionnaire and the body composition of adolescents (waist circumference, body fat percentage, and BMI); and (2) to analyze the association between PF (measured with the ALPHA-Fitness battery, AFB) and the body composition of adolescents (waist circumference, body fat percentage, and BMI).

## 2. Materials and Methods

The present study is a descriptive, quantitative, and cross-sectional research which followed the ethical standards defined by the Declaration of Helsinki (Hong Kong Review in September 1989 and Edinburgh 2000). It has been carried out in accordance with the recommendations for good clinical practices of the EC (Document 111/3976/88, July 1990). In order to meet the requirements for collecting data in the three participating cities, we needed the approval of the Ethics and Research Committee of Andalusia (Ref.: 0310-N-17), the National Bioethics Committee of Iceland (Ref.: VSNb2017030026/03.01) and the Ethics Committee of the University of Tartu, Estonia (Ref.: 281/T-10).

### 2.1. Study and Sample Design

The participants were students (N = 1717, 52.41% boys; 47.59% girls) from 13 to 16 years old (14.94 ± 1.12), enrolled in secondary schools in Seville (Spain), Reykjavik (Iceland), and Tartu (Estonia). For the selection of the sample, a 95% confidence interval and a 5% margin of error were applied.

### 2.2. Instruments

The Adherence to the Mediterranean Diet Questionnaire (KIDMED) had been previously validated in English and Spanish [18], and was handed to all the participants with statements in their mother tongue. For the Icelandic and Estonian versions parallel back-translation was used [20,21]. The KIDMED consists of 16 items (see Table 1). Twelve items represent a positive score for the adherence to the MD and the remaining 4 items represent a negative score. A positive answer to a question that involves greater adherence to the diet is worth +1 point. A positive answer to a question that means less adherence to the diet is worth −1 point. Negative answers do not score (a value of 0 is noted). The KIDMED index is the sum of all the scores and ranges from 0 to 12 points (minimum to maximum adherence to the MD). The adherence to the MD was categorized as: low adherence (very low-quality diet, 0–3); medium adherence (improvement of the diet is needed, 4–7); and high adherence (ideal adherence to the MD, 8–12).

Alpha Fitness Battery Test (AFB): The anthropometric variables of the participants (weight, height, BMI, fat percentage) and PF variables (cardiovascular endurance, upper body strength, lower body power, and speed/agility) were assessed using a modified version of the Extended Alpha fitness battery [22]. The measurement of the skin folds was omitted due to the limited time for carrying out the research and the large number of participants. Instead, a bioimpedance system (Tanita Inner Scan BF-689, Tanita, Tokyo, Japan) validated by the U.S. Food and Drug Administration for that type of population was used. When assessing PF and body composition parameters, the protocol established by the AFB was followed at all times. All the instruments used, and the procedures followed can be found in Galan-Lopez et al. (2018, 2019) as part of a larger research project [23,24].

### 2.3. Methodology

The measurements corresponding to the AFB and the data collection with the KIDMED questionnaire were carried out during the participants’ physical education classes. The physical tests were organized consecutively in a circuit. The cardiovascular endurance test was carried out by several students at the same time. The preparation, implementation and completion of all tests including the KIDMED questionnaire lasted 90 min for each group/class of participants.

### 2.4. Statistical Analysis

In order to present the data, percentages (%) and frequencies were used for the qualitative variables and the mean (M) ± standard deviations (SD) for the quantitative variables. After verifying the normality of the variables by the Kolmogorov-Smirnoff test, the Student’s *t*-test for independent samples was conducted in order to analyze age, height, and weight and to check possible differences between the KIDMED scores in boys and girls with over waist circumference (O_waist_), overfat (O_fat_), and overweight (O_weight_) and, without over waist circumference (N-O_waist_), overfat (N-O_fat_), and overweight (N-O_weight_). Additionally, a Chi-square test was performed for analyzing the proportion of participants with and without O_waist_, O_fat_, and O_weight_ who responded ‘yes’ or ‘no’ to the questions of the KIDMED questionnaire. Subsequently, to estimate the relative risk of responding positively to a risk factor in the questionnaire, an odd ratio (OR) was calculated. To analyze the PF performance in boys and girls with and without O_waist_, O_fat_, and O_weight_, the Student’s *t*-test for independent samples was performed. Since only European subjects took part in the present study and the AFB, developed and validated for European adolescent population was used, the different categories of waist perimeter, body fat percentage and BMI, were established based on the classification employed by the authors of the battery [25,26]. Cohen’s effect size (ES) was calculated to allow a better interpretation of the results related to these variables and the KIDMED index. The ES was considered as trivial (<0.2), small (0.2–0.5), moderate (0.5–0.8) and large (>0.8). The statistical significance was set at *p* < 0.05. All the statistical tests were performed using the software package SPSS version 24.0 (SPSS, Chicago, IL, USA, III).

## 3. Results

### 3.1. Descriptive Data in Subject Subjects with and without O_waist_, O_fat_, and O_weight_

**Figure 1:**
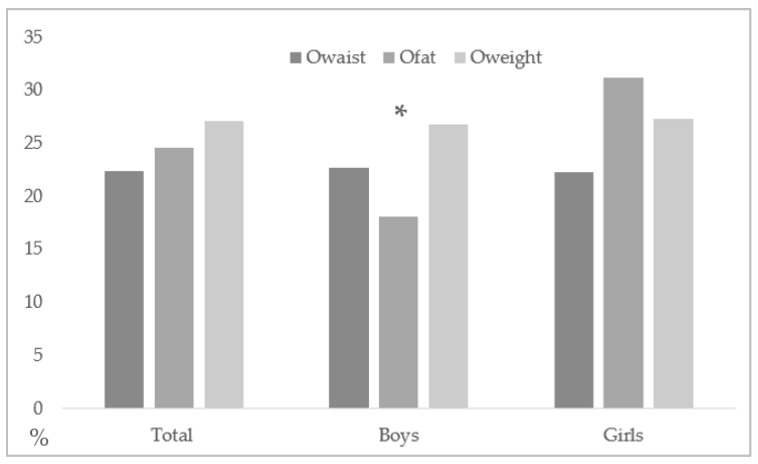
Total percentage of boys and girls (O_waist_, O_fat_, and O_weight_). * Statistical difference between boys and girls with O_fat_ (*p* < 0.05).

The average waist circumference of the sample is 72.76 ± 10.01 cm (75.17 ± 10.36 cm for boys and 70.12 ± 8.90 cm for girls), with a 22.4% (385/1717) rate of O_waist_ (22.7%, 204/900 in boys; 22.2%, 181/817 in girls). Body fat levels were 21.36 ± 8.62% (16.85 ± 7.72% in boys and 26.34 ± 6.58% in girls), with 24.6% (418/1717) for the rate of O_fat_ (18.1%, 163/900 in boys; 31.2%, 255/817 in girls) and a higher rate of O_fat_ in girls compared to boys (*p* < 0.001; OR = 1.41 (1.28–1.56)) was found. The BMI for the whole sample was 21.51 ± 4.05 kg/m^2^ (21.44 ± 4.09 kg/m^2^ in boys and 21.58 ± 4.02 kg/m^2^ in girls), with a 27.1% (464/1717) O_weight_ rate (26.8%, 241/900 in boys; 27.3%, 223/817 in girls) (see Figure 1).

**Figure 2:**
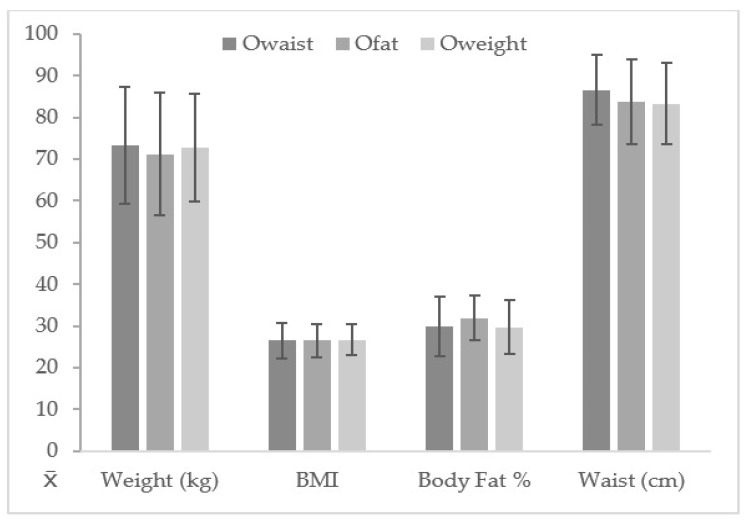
Body composition parameters according to O_waist_, O_fat_, and O_weight_ groups.

Table 2 shows that there were no statistically significant differences in relation to age between boys and girls with or without O_waist_, O_fat_, and O_weight_ (*p* > 0.05). Significant differences were found between boys and girls with O_waist_ in relation to height, showing a higher height for N-O_waist_ (*p* < 0.001; ES = 0.39; *p* = 0.002; ES = 0.27). In relation to weight, O_waist_, O_fat_, and O_weight_, the participants of both genders showed significantly higher results than the N-O_waist_, N-O_fat_, and N-O_weight_ participants (*p* < 0.001; ES:1.40–2.10). Also, higher levels of BMI (*p* < 0.001; ES: 1.83–2.98), body fat percentage (*p* < 0.001; ES: 1.73–3.38), and waist circumference (*p* < 0.001; ES: 1.85–2.84) were observed in O_waist_, O_fat_, and O_weight_ in comparison to the N-O_waist_, N-O_fat_, and N-O_weight_ subjects (see Figure 2).

### 3.2. KIDMED Index and Responses in Boys with and without O_waist_, O_fat_, and O_weight_

No statistically significant differences (*p* > 0.05) in relation to the KIDMED index according to the different body composition criteria used (O_waist_, O_fat_, and O_weight_) were found (see Table 2). When analyzing the positive (+) questions of the KIDMED (see Table 3), it was observed that there is a higher consumption of almost daily rice or pasta (≥5 times per week) in N-O_waist_ (*p* = 0.031), N-O_fat_ (*p* = 0.030), and N-O_weight_ (*p* = 0.041) participants. Thus, not eating rice or pasta almost daily (≥5 times per week) increases the risk of suffering from O_waist_ (OR = 1.34 (1.02–1.75)), O_fat_ (OR = 1.42 (1.04–1.94)), and O_weight_ (OR = 1.28 (1.01–1.63)). In turn, the N-O_waist_ participants had a higher regular fish consumption (2–3 times per week) (*p* = 0.029), hence being a risk factor not reaching that consumption rate (O = 1.31 (1.03–1.67)) and, more so, a second serving of fruit daily (*p* = 0.32), whose non-consumption increases the relative risk of O_waist_ (OR = 1.31(1.02–1.67)). N-O_weight_ participants also showed a superior regular nut consumption (≥2–3 times per week) (*p* = 0.014), thus not reaching such consumption increases the risk of O_weight_ (OR = 1.34 (1.06–1.69)).

Regarding the negative questions (−) (see Table 3), it was observed that skipping breakfast presented a higher value in participants with O_waist_ (*p* = 0.002), O_fat_ (*p* = 0.001), and O_weight_ (*p* = 0.001), considering this to be a risk factor for O_waist_ (OR = 1.48 (1.16–1.89)), O_fat_ (OR = 1.64 (1.24–2.16)), and O_weight_ (OR = 1.47 (1.19–1.83)) subjects. In addition, higher consumption of pastries and commercially baked goods for breakfast was found for the N-O_waist_ (*p* = 0.040), N-O_fat_ (*p* = 0.013), and N-O_weight_ (*p* = 0.025) participants, and a higher value for going to fast-food restaurants more than once per week in O_weight_ participants (*p* = 0.035). Finally, a higher consumption of sweets and candy several times per day was found in O_fat_ (*p* = 0.002).

### 3.3. KIDMED Responses in Girls with and without O_waist_, O_fat_ and O_weight_

No statistically significant differences were observed for the girls in relation to the KIDMED index according to the different body composition criteria used (O_waist_, O_fat_, and O_weight_ (*p* > 0.05) (see Table 2). Regarding the positive (+) questions of the KIDMED questionnaire (see Table 4), N-O_waist_ (*p* = 0.022) and N-O_weight_ (*p* = 0.026) girls had a higher consumption of dairy products for breakfast, considering non-consumption to be a risk factor for O_waist_ (OR = 1.38 (1.06–1.80)) and O_weight_ (OR = 1.33 (1.05–1.68)). As for the boys, a higher consumption of almost daily rice or pasta (≥5 times per week) was observed in N-O_waist_ girls (*p* = 0.008), assuming this to be a risk factor for O_waist_ (OR = 1.49 (1.10–2.01)) if they did not consume them so frequently. Unlike what happened for O_waist_ boys, significant differences had been found for “second serving of fruit daily” with a higher consumption in O_waist_ (*p* = 0.003), O_fat_ (*p* = 0.005) and O_weight_ girls (*p* = 0.034). So, not consuming a second serving of fruit daily implies a risk factor in the development of O_waist_ (OR = 0.68 (0.52–0.88)), O_fat_ (OR = 0.74 (0.61–0.91)), and O_weight_ (OR = 0.78 (0.62–0.98)) in girls. Also, there has been a tendency toward a greater intake of cereal or grains products for breakfast in N-O_weight_ girls (*p* = 0.066), increasing the risk of O_weight_ (OR = 1.26 (1.00–1.59)) if not consumed.

Regarding the negative questions of the questionnaire (−) (see Table 4), a lower intake of sweets and candy every day was observed in O_waist_ (*p* = 0.001), O_fat_ (*p* = 0.006), and O_weight_ girls (*p* = 0.006). Likewise, the N-O_waist_ and N-O_fat_ girls go less to fast-food restaurants in comparison to O_waist_ (*p* = 0.005) and O_fat_ (*p* = 0.030).

### 3.4. Fitness Performance in Boys and Girls with and without O_waist_, O_fat_ and O_weight_

**Figure 3:**
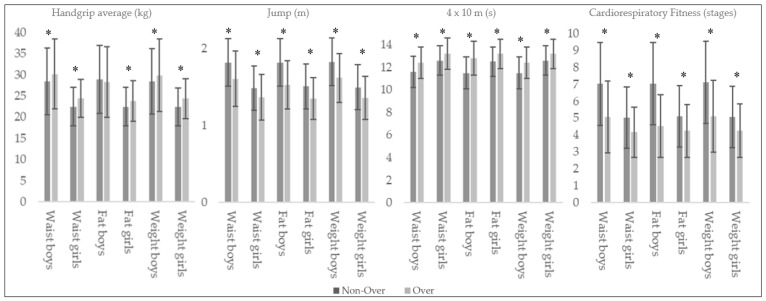
Physical fitness values in over and non-over boys and girls according body composition parameters. * Statistical difference between groups (*p* < 0.05).

In handgrip performance, higher values for O_fat_, O_waist_, and O_weight_ (*p* < 0.05) were found except for the O_fat_ boys (*p* = 0.359) (see Table 5). The jump test showed a higher significant performance and a moderate effect size for N-O_waist_, N-O_fat_, and N-O_weight_ girls (*p* < 0.001; ES = 0.41–0.56), and moderate to large in the case of N-O_waist_, N-O_fat_, and N-O_weight_ boys (*p* < 0.001; ES = 0.65-0.94). In the case of the speed test, higher values have been verified with a moderate effect size for N-O_waist_, N-O_fat_, and N-O_weight_ girls (*p* < 0.05; ES = 0.45–0.54), and moderate and large values in boys (*p* < 0.001; ES = 0.57–0.92). Likewise, in cardiorespiratory fitness, N-O_waist_, N-O_fat_, and N-O_weight_ boys obtained a statistically superior performance with a large effect size (*p* < 0.001; ES = 0.83–1.08), while those statistically significant differences in girls had a moderate effect size (*p* < 0.001; ES = 0.47–0.50) (see Figure 3).

## 4. Discussion

### 4.1. Descriptive Data in Subjects with and without O_waist_, O_fat_, and O_weight_

Current data denote 124 million children and adolescents with adiposity (>5–19 years old). This number has increased tenfold in the last 30 years [3]. Obesity rates in adolescents of the countries participating in this study are 8.5% in Spain and Iceland and 5% in Estonia, as one of the countries with the lowest rate of obesity [3]. If we take into account the results regarding the BMI of the overweight and obese subjects of the present sample, it can be seen how these are slightly higher than those obtained in studies with a similar population (27.04% vs. 21–23%) [27,28,29,30,31,32], although both are in line with the increase and prevalence of overweight and current obesity in the adolescent population [3].

Despite the widespread use of BMI as an indicator of adiposity in the population, its correlation with body fat is relatively poor, given that it shows little sensitivity when determining the different deposits of fat, mainly abdominal, due to its extensive relationship with noncommunicable diseases like obesity [4]. In order to correct this aspect, the BMI was complemented with the waist circumference and the body fat percentage. It can be observed how the results regarding waist circumference are similar to those obtained in various studies on European adolescent populations (21.51 cm vs. 21.1–21.7 cm) [27,28,31,33,34]. Regarding the percentage of body fat, the results show a great similarity with those obtained in several investigations (21.36% vs. 22–22.7%), although these were obtained by measuring the skin folds [28,29,33]. In addition, there is a higher O_fat_ rate in girls than boys. Although this aspect would need to be gone into more thoroughly, a possible cause could be a lower level of PA and a more sedentary lifestyle in girls at these ages [35,36].

It is worth highlighting the existence of significant differences in all the body composition variables for both genders in the O_waist_, O_fat_, and O_weight_ subjects in comparison to the N-O_waist_, N-O_fat_, and N-O_weight_ subjects. This supports the classification used in the present investigation.

### 4.2. KIDMED Index and Responses in Boys and Girl with and without O_waist_, O_fat_, and O_weight_

Adolescence is a key period in life, and it involves multiple physiological and psychological changes that affect nutritional needs and habits. Teenagers have different choices and eating habits compared to children and adults [37]. In this aspect, the MD is revealed to be an appropriate dietary pattern [17]. Most recently reviewed studies conducted in southern European countries reported that approximately half the children and adolescents show a low adherence to MD [38]. The participants of the current study presented a medium level of adherence to the MD (5–6) [39]. These values corroborate the aforementioned and coincide with other, similar studies carried out in adolescents in southern Europe [40,41].

Recent research suggests that the main benefits derived from the implementation or monitoring of the MD translate into an improvement in body composition [42]. The answers to the positive (+) questions of the KIDMED and its relationship with the parameters of body composition according to sex show heterogeneity, though there are results that merit analyzing. Thus, a greater consumption of rice or pasta, almost daily, (≥5/week) can be observed in N-O_waist_ individuals (boys and girls), N-O_fat_ (boys), and N-O_weight_ (boys), so not consuming them with that frequency could be a risk factor in this sample. As question Q8 does not discriminate between brown and refined rice or pasta, the results may lead to different interpretations. On the one hand, an excessive consumption of refined carbohydrates is negatively related to health and obesity [43,44,45,46] and, on the other hand, the consumption of wholegrain carbohydrates in adolescents is positively related to both [47,48,49,50].

When analyzing the N-O_waist_ participants, a higher regular fish consumption (2–3/week) is observed in boys and, a greater consumption of dairy products for breakfast in girls. So, not consuming them could be a risk factor in both cases. The consumption of fish and dairy products is a fundamental aspect in the MD [39]. Numerous systematic reviews and meta-analyses attribute a lower incidence of diseases and a better health to fish consumption [51,52], but few relate this to a better body composition in adolescents. Although the consumption of fish, together with vegetables and fruit, seems to be a pending task in adolescence, the results found for N-O_waist_ boys coincide with similar studies where children and adolescents had a regular consumption of fish [35,38]. On the other hand, although more research is needed to examine the types of dairy products in relation to the risk of childhood and adolescent overweight, the accumulated evidence from studies suggests that dairy consumption is associated inversely and longitudinally with the risk of overweight and obesity in these ages [53,54]. In addition, having them for breakfast reduces the chances of choosing less healthy products [55,56].

In the case of the N-O_weight_ participants, a regular consumption of nuts (≥2–3/week) is observed in boys. The impact of a regular consumption of nuts on health is more than proven [57]. In addition, its consumption is inversely related to the risk of overweight and obesity [58], data that support the results obtained in this group. As in the N-O_waist_ girls, the N-O_weight_ girls had a higher consumption of dairy products and cereal or grain products for breakfast. Both aspects support a better choice of food for breakfast by these two groups of girls, being therefore able to condition a better body composition, since not having these habits in the case of O_waist_ and O_weight_ in the present sample turned out to be a risk factor.

Having a second serving of fruit daily is controversial between boys and girls, since a greater consumption in N-O_waist_ boys could increase the relative risk of O_waist_, but there is a greater consumption in O_waist_, O_fat_, and O_weight_ girls, in whom, however, not consuming it prevents the development of O_waist_, O_fat_, and O_weight_ in them. Although non-self-reported studies are necessary to be more conclusive in this regard, a possible explanation could be the lack of concretion of Q1 and Q2, in which fruit and fruit juice are matched. This could, in the worst case, be from industrial production. This aspect is fundamental, since the degree of food processing influences the health effects of fruit-based products [59,60]. Thus, whole fresh fruit does not contribute to obesity and may have a place in the prevention and management of excess adiposity [59,61,62], 100% fruit juices can have intermediate effects [63], and canned fruit and sweetened fruit juice are positively associated with the risk of all-cause mortality and type 2 diabetes, as well as obesity [59,60,61]. This aspect is important in order to adjust the official recommendations for fruit consumption.

The answers to the negative (−) questions of the KIDMED questionnaire and their relation to the parameters of body composition analyzed according to sex also offered disparities. Ultra-processed, hypercaloric and high-fat food products, sugar, and added salt are one of the main factors that cause obesity in these ages [64,65,66,67]. This is related to some of the results obtained in boys, where there is a higher consumption of sweets and candy several times every day in O_fat_ or more visits to fast-food restaurants (>1/week) in O_weight_ participants. These results coincide with similar studies in adolescents, where daily intake of sweets or going to fast-food restaurants more than once was common [38,39,68].

On the contrary, other results diverge with a lower consumption of pastries and commercially baked goods for breakfast in O_waist_, O_fat_, and O_weight_ boys, or a lower level of intake of sweets and candy several times every day in O_waist_, O_fat_, and O_weight_ girls. In addition, these contradictory data are reinforced by verifying that the O_waist_ and O_fat_ girls go less frequently to fast-food restaurants. Although the choice of healthy foods is crucial at these ages, impacting on their current and future health [69], more thorough studies that relate this aspect to body composition beyond the BMI are required in adolescents.

Breakfast has been associated with a lower risk of overweight and obesity in children and adolescents [70,71]. Moreover, skipping breakfast correlates with a worse body composition in both [37,68]. The results obtained in the present study are in line with both aspects, since skipping breakfast was more common in O_waist,_ O_fat_, and O_weight_ boys, and could be also considered as a risk factor in this sample. This is currently still a controversial topic, since despite the number of studies and reviews that support it, the evidence reviewed does not confirm a causal relationship, due to the quality, the poor design of some studies, or the possible influence of confounding variables [72,73]. We need to keep in mind that much of the food that teenagers usually eat for breakfast is quite unhealthy [68] and that, therefore, it is more important to focus the debate on the quality of breakfast food, rather than on the dichotomy: having breakfast or not.

The lack of information about solid approaches to nutrition, the intensive promotion of ultra-processed food and beverages at these ages, as well as the limited availability of adequate nutritional education contribute to aggravating the problem of food at this age stage [74].

### 4.3. Fitness Performance in Boys and Girls with and without O_waist_, O_fat_, and O_weight_

A high level of fitness from childhood to adulthood is positively related to health and a key factor in maintaining the current and future health status [75,76]. Moreover, abdominal adiposity in combination with a poor PF are directly associated with serious diseases [77,78]. The association between body composition parameters (waist circumference, percentage of body fat, and BMI) and performance in numerous fitness tests has been extensively studied in Europe [79] and non-European countries [80,81], thus determining the need for preventive work and the monitoring of the health status of adolescents. If we examine the results of the tests of the AFB, a better performance can be observed among those participants with N-O_waist_, N-O_fat_, and N-O_weight_ in the long jump, speed/agility, and cardiorespiratory resistance tests. These data are supported by various research studies that related elements of body composition and performance in various fitness tests [82,83,84]. The lower performance of O_waist_, O_fat_, and O_weight_ subjects could be due to their excess of weight since these tests involve propulsion or lifting of their own body mass [23,29]. Anyway, the low PF of the O_waist_, O_fat_ and O_weight_ adolescents could condition an early onset of diseases that in turn would be aggravated by their early overweight.

On the other hand, manual dynamometry is the only test in which the O_waist_, O_fat_, and O_weight_ subjects of both sexes have higher results, except for O_fat_ boys. Similar data have been found by Gulias-Gonzalez et al. (2014) [85] and Garcia-Pastor et al. (2016) [83]. Both studies showed that overweight participants had better results in the hand grip test. Taking into account our measurements of waist circumference, the BMI and percentage of body fat, the highest values in the manual dynamometry test could be justified by the different rates of growth, bone and muscle development found in overweight and obese adolescents [86].

In addition, it should be borne in mind that overweight and obese adolescents have lower levels of participation in sports activities, with a tendency to be less active than those adolescents with a normal body composition [87]. This predisposes them to increase their obesity levels and reduce PF levels [88].

This study’s cross-sectional design has limitations, as the contributions must not be attributed to plausible causes. These could be used as indications for forthcoming research works. In addition, the data collection using the KIDMED questionnaire was self-reported, which could lead to an error in the reports and to memory bias due to the nature of the study.

## 5. Conclusions

The values of O_waist_, O_fat_, and O_weight_ ranged from 21.4% (O_waist_) to 27.1% (O_weight_) with gender differences only in O_fat_, in which the prevalence is higher in girls than boys. The O_waist_, O_fat_, and O_weight_ participants of both sexes presented higher levels of body mass, waist circumference, body fat percentage and BMI. No significant differences were found in the KIDMED Index for the N-O_waist_, N-O_fat_, and N-O_weight_ groups, although differences have been observed in some responses to KIDMED (i.e., consumes rice or pasta almost daily, skips breakfast, has pastries or commercially baked goods for breakfast in boys, has a second serving of fruit daily, eats sweets and candy several times every day, goes more than once per week to a fast-food restaurant, and eats dairy products for breakfast). Some of these differences could represent a risk factor for the condition of O_waist_, O_fat_, and O_weight_ in the present sample.

Although it seems that the adherence to the MD measured with KIDMED does not have a great influence on the condition of O_waist_, O_fat_, and O_weight_, it has been proven that there is a direct relationship between PF and O_waist_, O_fat_, and O_weight_ as boys and girls showed significantly lower results in all the AFB tests, except for the handgrip test.

The differences between the N-O_waist_, N-O_fat_, and N-O_weight_ vs. O_waist_, O_fat_, and O_weight_ groups in relation to the variables analyzed highlight the need to make institutional and educational efforts aimed at promoting healthy eating habits and effective programs for physical exercise.

## Figures and Tables

**Table 1:** Adherence to the Mediterranean Diet Questionnaire (KIDMED).

Q1. Takes a fruit or fruit juice daily (+)	Q9. Has cereal or grains product for breakfast (+)
Q2. Has a second serving of fruit daily (+)	Q10. Regular nut consumption (≥2–3/week) (+)
Q3. Has fresh or cooked vegetables daily (+)	Q11. Uses of olive oil at home (+)
Q4. Has fresh or cooked vegetables more than 1/day (+)	Q12. Skips breakfast (−)
Q5. Regular fish consumption (at least 2–3/week) (+)	Q13. Has dairy product for breakfast (+)
Q6. Goes >1/week fast food restaurant (−)	Q14. Pastries/Commercially baked goods for breakfast (−)
Q7. Likes Pulses and eats more than 1/week (+)	Q15. Two yoghurts and/or 40 g cheese daily (+)
Q8. Consumes rice or pasta almost daily (≥5/week) (+)	Q16. Takes sweets and candy several times every day (−)

**Table 2:** Data for age, height, weight, and KIDMED index in over and non-over boys and girls according to different criteria selected.

Variable	Gender	Waist				Percentage of Body Fat				BMI			
		N-O_waist_	O_waist_	*p*-Value	ES	N-O_fat_	O_fat_	*p*-Value	ES	N-O_weight_	O_weight_	*p*-Value	ES
Age (years)	Boys	14.98 ± 1.12	14.85 ± 1.18	0.142	0.11	14.98 ± 1.12	14.81 ± 1.18	0.82	0.15	14.99 ± 1.12	14.84 ± 1.17	0.070	0.13
	Girls	14.93 ± 1.12	14.96 ± 1.10	0.783	0.03	14.93 ± 1.14	14.96 ± 1.06	0.713	0.03	14.86 ± 1.11	15.03 ± 1.10	0.072	0.15
Height (m)	Boys	1.67 ± 0.11	1.70 ± 0.11	0.002 *	0.27	1.68 ± 0.11	1.66 ± 0.11	0.052	0.18	1.68 ± 0.11	1.68 ± 0.10	0.288	0.00
	Girls	1.60 ± 0.08	1.63 ± 0.07	<0.001 *	0.39	1.61 ± 0.08	1.60 ± 0.07	0.453	0.13	1.60 ± 0.08	1.61 ± 0.07	0.282	0.13
Weight (kg)	Boys	56.13 ± 10.88	77.30 ± 15.70	<0.001 *	1.75	57.57 ± 12.09	76.12 ± 17.48	<0.001 *	1.4	55.25 ± 10.37	76.43 ± 14.87	<0.001 *	1.81
	Girls	52.02 ± 8.30	69.36 ± 12.10	<0.001 *	1.87	51.11 ± 8.02	66.32 ± 11.87	<0.001 *	1.62	50.95 ± 7.43	68.94 ± 11.06	<0.001 *	2.1
Body mass index (kg/m^2^)	Boys	19.92 ± 2.55	26.64 ± 4.11	<0.001 *	2.26	20.16 ± 2.26	27.23 ± 4.16	<0.001 *	2.62	19.51 ± 2.09	26.74 ± 3.50	<0.001 *	2.84
	Girls	20.24 ± 2.57	26.32 ± 4.58	<0.001 *	1.95	19.69 ± 2.19	25.76 ± 4.01	<0.001 *	2.11	19.72 ± 2.00	26.56 ± 3.82	<0.001 *	2.61
Body fat (%)	Boys	14.22 ± 5.18	25.82 ± 8.25	<0.001 *	1.93	13.97 ± 4.43	29.87 ± 5.82	<0.001 *	3.38	13.64 ± 4.64	25.62 ± 7.69	<0.001 *	2.13
	Girls	24.22 ± 5.03	33.82 ± 5.95	<0.001 *	1.83	22.96 ± 3.94	33.81 ± 4.88	<0.001 *	2.55	23.55 ± 4.49	33.79 ± 5.39	<0.001 *	2.16
Waist (cm)	Boys	70.80 ± 5.73	90.01 ± 8.76	<0.001 *	2.94	72.07 ± 7.17	89.11 ± 11.09	<0.001 *	2.13	70.72 ± 6.02	87.28 ± 10.06	<0.001 *	2.26
	Girls	66.40 ± 4.70	83.09 ± 8.01	<0.001 *	2.98	66.35 ± 5.50	78.36 ± 9.42	<0.001 *	1.73	66.63 ± 5.69	79.36 ± 9.33	<0.001 *	1.85

Data presented as M ± SD. * Statistical difference between groups (*p* < 0.05). ES: effect size. O_waist_: over waist circumference; O_fat_: overfat; O_weight_: overweight; N-O_waist_: non-over waist circumference; N-O_fat_: non-overfat; N-O_weight_: non-overweight.

**Table 3:** Responses in the KIDMED questionnaire in over and non-over boys according to selected body composition parameters.

KIDMED Question	R	Waist				Percentage of Body Fat				BMI			
		N-O_waist_ (*n* = 696)	O_waist_ (*n* = 204)	*p*-Value	OR	N-O_fat_ (*n* = 737)	O_fat_ (*n* = 163)	*p*-Value	OR	N-O_weight_ (*n* = 659)	O_weight_ (*n* = 241)	*p*-Value	OR
Q1_(+)_	Yes	70.5%	64.2%	0.101	1.25 (0.97–1.60)	69.9%	65.6%	0.303	1.17 (0.88–1.57)	70.7%	64.7%	0.088	1.22 (0.98–1.52)
Q2_(+)_	Yes	50.7%	42.2%	0.032 *	1.31 (1.02–1.67)	49.84%	44.2%	0.196	1.20 (0.91–1.59)	50.5%	44.0%	0.084	1.21 (0.98–1.51)
Q3_(+)_	Yes	50.4%	57.4%	0.094	0.81 (0.631–1.03)	52.4%	50.3%	0.665	1.07 (0.81–1.41)	50.7%	55.6%	0.201	0.87 (0.70–1.08)
Q4_(+)_	Yes	28.2%	34.8%	0.081	0.79 (0.62–1.02)	29.3%	31.3%	0.636	0.93 (0.69–1.25)	28.1%	34.0%	0.084	0.82 (0.65–1.03)
Q5_(+)_	Yes	60.6%	52.0%	0.029 *	1.31 (1.03–1.67)	58.6%	58.9%	1.00	0.99 (0.75–1.31)	59.5%	56.4%	0.445	1.10 (0.88–1.36)
Q6_(−)_	Yes	32.6%	27.9%	0.231	0.84 (0.64–1.10)	32.7%	26.4%	0.136	0.77 (0.57–1.07)	33.5%	26.1%	0.035 *	0.77 (0.60–0.99)
Q7_(+)_	Yes	48.7%	50.5%	0.691	0.95 (0.74–1.21)	47.9%	54.6%	0.141	0.80 (0.61–1.06)	47.2%	54.4%	0.060	0.81 (0.65–1.00)
Q8_(+)_	Yes	37.6%	29.4%	0.031 *	1.34 (1.02–1.75)	37.4%	28.2%	0.030 *	1.42 (1.04–1.94)	37.8%	30.3%	0.041 *	1.28 (1.01–1.63)
Q9_(+)_	Yes	80.2%	78.9%	0.692	1.06 (0.79–1.42)	80.7%	76.1%	0.195	1.25 (0.91–1.72)	79.8%	80.1%	1.00	0.99 (0.75–1.30)
Q10_(+)_	Yes	40.2%	38.7%	0.745	1.05 (0.82–1.35)	41.1%	34.4%	0.113	1.27 (0.95–1.70)	42.3%	33.2%	0.014 *	1.34 (1.06–1.69)
Q11_(+)_	Yes	80.6%	79.4%	0.690	1.06 (0.79–1.43)	79.8%	82.8%	0.446	0.85 (0.58–1.23)	80.6%	79.7%	0.777	1.04 (0.80–1.36)
Q12_(−)_	Yes	27.3%	38.7%	0.002 *	1.48 (1.16–1.89)	27.4%	41.1%	0.001 *	1.64 (1.24–2.16)	26.7%	38.6%	0.001 *	1.47 (1.19–1.83)
Q13_(+)_	Yes	80.3%	75.0%	0.117	1.26 (0.96–1.66)	79.4%	77.9%	0.671	1.07 (0.77–1.50)	79.7%	77.6%	0.517	1.09 (0.85–1.41)
Q14_(−)_	Yes	34.6%	27.0%	0.040 *	0.75 (0.57–0.99)	34.7%	24.5%	0.013 *	0.66 (0.48–0.92)	35.1%	27.0%	0.025 *	0.75 (0.59–0.97)
Q15_(+)_	Yes	46.7%	43.1%	0.381	1.12 (0.88–1.43)	46.1%	44.8%	0.795	1.05 (0.79–1.38)	45.5%	46.9%	0.763	0.96 (0.77–1.19)
Q16_(−)_	Yes	23.7%	19.1%	0.184	0.81 (0.59–1.10)	24.7%	13.5%	0.002 *	0.53 (0.35–0.81)	24.3%	18.3%	0.059	0.76 (0.57–1.02)

Data presented as percentages (%) and frequencies; * Statistical difference between groups (*p* < 0.05). R = response. O_waist_: over waist circumference; O_fat_: overfat; O_weight_: overweight; N-O_waist_: non-over waist circumference; N-O_fat_: non-overfat; N-O_weight_: non-overweight.

**Table 4:** Responses in the KIDMED questionnaire in over and non-over girls according to selected body composition parameters.

KIDMED Question	R	Waist				Percentage of Body Fat				BMI			
		N-O_waist_ (*n* = 636)	O_waist_ (*n* = 181)	*p*-Value	OR	N-O_fat_ (*n* = 562)	O_fat_ (*n* = 255)	*p*-Value	OR	N-O_weight_ (*n* = 594)	O_weight_ (*n* = 223)	*p*-Value	OR
Q1_(+)_	Yes	69.2%	75.1%	0.139	0.79 (0.59–1.07)	69.2%	73.3%	0.247	0.87 (0.69–1.10)	70.0%	71.7%	0.667	0.94 (0.73–1.21)
Q2_(+)_	Yes	44.3%	56.9%	0.003 *	0.68 (0.52–0.88)	43.8%	54.5%	0.005 *	0.74 (0.61–0.91)	44.8%	53.4%	0.034 *	0.78 (0.62–0.98)
Q3_(+)_	Yes	60.4%	59.1%	0.797	1.04 (0.80–1.35)	58.9%	62.7%	0.317	0.89 (0.72–1.11)	59.4%	61.9%	0.575	0.93 (0.74–1.17)
Q4_(+)_	Yes	33.8%	35.9%	0.596	0.93 (0.71–1.22)	33.3%	36.5%	0.382	0.91 (0.74–1.12)	33.2%	37.2%	0.283	0.88 (0.70–1.11)
Q5_(+)_	Yes	59.4%	59.7%	1.00	0.99 (0.76–1.29)	57.8%	63.1%	0.166	0.86 (0.69–1.06)	58.9%	61.0%	0.631	0.94 (0.75–1.18)
Q6_(−)_	Yes	31.0%	20.4%	0.005 *	0.64 (0.46–0.89)	31.0%	23.5%	0.030 *	0.77 (0.60–0.98)	29.8%	25.6%	0.259	0.86 (0.66–1.10)
Q7_(+)_	Yes	50.6%	48.6%	0.674	1.07 (0.82–1.38)	49.5%	51.8%	0.547	0.94 (0.77–1.15)	49.5%	52.0%	0.531	0.93 (0.74–1.16)
Q8_(+)_	Yes	36.6%	26.0%	0.008 *	1.49 (1.10–2.01)	35.8%	31.0%	0.203	1.16 (0.93–1.45)	36.0%	29.6%	0.098	1.24 (0.97–1.59)
Q9_(+)_	Yes	73.6%	66.9%	0.091	1.28 (0.98–1.68)	73.8%	68.2%	0.110	1.20 (0.97–1.49)	73.9%	67.3%	0.066	1.26 (1.00–1.59)
Q10_(+)_	Yes	33.8%	32.0%	0.721	1.06 (0.81–1.40)	32.4%	35.7%	0.379	0.90 (0.73–1.11)	33.8%	32.3%	0.739	1.05 (0.83–1.34)
Q11_(+)_	Yes	83.2%	84.5%	0.734	0.93 (0.65–1.32)	82.4%	85.9%	0.224	0.83 (0.62–1.12)	83.0%	84.8%	0.598	0.91 (0.66–1.25)
Q12_(−)_	Yes	36.8%	40.9%	0.339	1.14 (0.88–1.48)	35.6%	108%	0.073	1.21 (0.99–1.49)	36.0%	42.2%	0.124	1.20 (0.96–1.51)
Q13_(+)_	Yes	75.5%	66.9%	0.022 *	1.38 (1.06–1.80)	75.3%	69.8%	0.104	1.20 (0.97–1.50)	75.8%	67.7%	0.026 *	1.33 (1.05–1.68)
Q14_(−)_	Yes	31.4%	23.8%	0.053	0.74 (0.54–1.00)	31.5%	25.9%	0.117	0.83 (0.65–1.05)	31.0%	26.5%	0.229	0.85 (0.66–1.10)
Q15_(+)_	Yes	37.4%	33.7%	0.383	1.14 (0.86–1.49)	37.4%	34.9%	0.531	1.08 (0.87–1.33)	37.9%	33.2%	0.222	1.16 (0.92–1.48)
Q16_(−)_	Yes	27.4%	14.9%	0.001 *	0.54 (0.37–0.78)	27.4%	18.4%	0.006 *	0.69 (0.53–0.91)	27.1%	17.9%	0.006 *	0.67 (0.50–0.91)

Data presented as like percentages (%) and frequencies; * Statistical difference between groups (*p* < 0.05). R = response. O_waist_: over waist circumference; O_fat_: overfat; O_weight_: overweight; N-O_waist_: non-over waist circumference; N-O_fat_: non-overfat; N-O_weight_: non-overweight.

**Table 5:** Physical fitness values in over and non-over boys and girls according to selected body composition parameters.

A-F Variable	Gender	Waist				Percentage of Body Fat				BMI			
		N-O_waist_	O_waist_	*p*-Value	ES	N-O_fat_	O_fat_	*p*-Value	ES	N-O_weight_	O_weight_	*p*-Value	ES
Handgrip average (kg)	Boys	28.4 ± 7.9	30.2 ± 8.3	0.005 *	0.23	28.9 ± 8.0	28.3 ± 8.3	0.359	0.07	28.4 ± 7.7	29.9 ± 8.6	0.014 *	0.19
	Girls	22.5 ± 4.6	24.4 ± 4.5	<0.001 *	0.42	22.5 ± 4.5	23.8 ± 4.8	<0.001 *	0.07	22.4 ± 4.5	24.4 ± 4.7	<0.001 *	0.44
Jump (m)	Boys	1.82 ± 0.31	1.61 ± 0.36	<0.001 *	0.65	1.82 ± 0.31	1.53 ± 0.31	<0.001 *	0.94	1.83 ± 0.31	1.62 ± 0.32	<0.001 *	0.67
	Girls	1.49 ± 0.29	1.37 ± 0.30	<0.001 *	0.41	1.51 ± 0.29	1.35 ± 0.27	<0.001 *	0.56	1.50 ± 0.29	1.36 ± 0.28	<0.001 *	0.49
4 × 10 m (s)	Boys	11.6 ± 1.4	12.4 ± 1.4	<0.001 *	0.57	11.5 ± 1.4	12.8 ± 1.5	<0.001 *	0.92	11.5 ± 1.4	12.4 ± 1.4	<0.001 *	0.64
	Girls	12.6 ± 1.3	13.2 ± 1.4	<0.001 *	0.45	12.5 ± 1.3	13.2 ± 1.3	0.030 *	0.54	12.6 ± 1.3	13.2 ± 1.3	<0.001 *	0.46
Cardiorespiratory Fitness (stages)	Boys	7.03 ± 2.45	5.06 ± 2.11	<0.001 *	0.83	7.04 ± 2.42	4.54 ± 1.85	<0.001 *	1.08	7.12 ± 2.43	5.11 ± 2.13	<0.001 *	0.85
	Girls	5.03 ± 1.81	4.17 ± 1.48	<0.001 *	0.49	5.11 ± 1.81	4.25 ± 1.56	<0.001 *	0.50	5.06 ± 1.80	4.24 ± 1.58	<0.001 *	0.47

Data presented as M ± SD; * Statistical difference between groups (*p* < 0.05). O_waist_: over waist circumference; O_fat_: overfat; O_weight_: overweight; N-O_waist_: non-over waist circumference; N-O_fat_: non-overfat; N-O_weight_: non-overweight.
